# Treatment of Hay-Fever—Emetine in Tuberculosis

**Published:** 1914-05-23

**Authors:** 


### MODERN METHODS SERIES.

### Treatment of Hay-Fever—Emetine in Tuberculosis.

It seems possible that the modern school of bacterial immunisation will score one of its greatest triumphs in the treatment of hay-fever. For several years laborious experiments have been going on, notably at St. Mary's Hospital, with a view to determining in what direction the principles of the new school of bacteriological therapy could best be applied in the relief of those distressing catarrhs that occasion so much suffering in the spring and summer. The late Mr. Leonard Noon, an old "Bart.'s" student who joined Sir Almroth Wright's band of investigators some seven or eight years ago, was largely instrumental in putting the hay-fever research upon a scientific basis, although he never reaped the reward of his work in this field, as he was unfortunately stricken with a fatal illness before he had time to put his conclusions to the practical test. However, one of his colleagues, Dr. John Freeman, carried on the work so ably begun by Mr. Noon.

Throughout, the attempt has been made to increase the bodily resistance to the pollen toxin by inoculations with vaccines prepared from grass pollens, chief attention having been given to the seed-dust of *Phleum pratense*, and hopeful reports have been made as to the results of treatment conducted on these lines. But nothing so encouraging has ever been published in regard to the treatment of hay-fever as the summary of results obtained during the last three years just reported to the *Lancet* (April 25, 1914) by Dr. Freeman; nor has any investigator ever been able to come to such a satisfactory conclusion in regard to his method as this investigator, who concludes his paper by a definite statement that "hay-fever treatment by active immunisation with a pollen vaccine, whether judged by statistics or by the experimental method, has succeeded, and the immunity thus acquired seems to last for one year at least after treatment has been discontinued."

### The Relief by Vaccine Treatment.

In the result, concerning the particular research just referred to, eighty-four cases of hay-fever were treated, who, after or during the treatment, between them went through 113 hay-fever seasons; and taking these seasons as the basis of analysis, it was found that in just over 30 per cent. the illness was completely or practically cured; whilst in 11.5 per cent. the catarrh was not improved, amelioration to a greater or less extent being exhibited in the remaining, approximately, 58 per cent. of seasons. As Dr. Freeman points out, no figures can give as clear a picture of the relief given by vaccine treatment in hay-fever as the report of one or two of the worst cases. Thus he relates an instance in which a patient was so incapacitated every year by spring catarrh that summer after summer he was forced to give up the work on which he depended to go for a sea voyage. In this case the symptoms were so severe that not only was the conjunctiva peeled off in places through the acute inflammation, but constant dribbling of tears severely excoriated the cheeks, and ice-bags had to be applied to relieve the intense pain in the eyes. After nineteen years of suffering in this way, a long treatment by inoculation, carried out through a whole winter and subsequent spring, brought him such relief that he became quite cured and even able to go through hay-fields with complete immunity. The success of the treatment appears to depend on its being commenced as early in the year as possible, and being systematically followed up; whilst patients who go to the time, trouble, and expense of having this treatment carried out, have the satisfaction of knowing that, so far as can be at present judged, the cure once obtained is lasting.

### Emetine in Tuberculosis.

A report by Dr. James Raeburn, of the Battersea Dispensary for the Prevention of Consumption, on the use of subcutaneous injections of emetine in pulmonary tuberculosis deserves the attention of medical practitioners. Using emetine in various types of cases, this investigator, in reporting his results to the *British Medical Journal* (March 28, 1914), is able to state that the drug not only relieves concomitant bronchitis, but appears to have a direct action in improving the condition of the lungs. Particularly is it noted that this compound is beneficial in the congestive stage of the disease, and in one of the cases quoted, in which tuberculin injections had failed, emetine given twice weekly for four weeks brought about a notable improvement.

It appears, moreover, that Dr. Raeburn's attention was first attracted to this method through the work of two French investigators, Drs. Flandin and Joltrain, who have found the emetine injections useful in stopping the hæmorrhage of phthisis; he is inclined to exceed the dosage in use abroad, where each injection contains 0.04 cg. In this country emetine can be obtained in ampules containing $\frac{1}{2}$ gm. of the drug in 15 minims of distilled water; and it is recommended that this be diluted with four parts of water, so that one minim holds in solution 0.04 cg. of emetine. Four minims of this dilute solution are said to be a suitable dose where it is desired to diminish expectoration or congestion. Cardiac weakness is to be considered a contra-indication to the treatment. Apparently where tubercle bacilli are present in the sputum, evidencing advanced disease, the treatment is not efficacious, and Dr. Raeburn considers that emetine has probably no direct anti-bacterial action. However, if his results as to the efficacy of emetine in the relief of pulmonary congestion be confirmed, it is quite possible that the introduction of this drug will be found to have given us an important adjunct to the routine treatment of consumption.

